# mHealth Intervention for Improving Pain, Quality of Life, and Functional Disability in Patients With Chronic Pain: Systematic Review

**DOI:** 10.2196/40844

**Published:** 2023-02-02

**Authors:** Marta Moreno-Ligero, Jose A Moral-Munoz, Alejandro Salazar, Inmaculada Failde

**Affiliations:** 1 Preventive Medicine and Public Health Area Department of Biomedicine, Biotechnology and Public Health University of Cádiz Cádiz Spain; 2 Observatory of Pain University of Cádiz Cádiz Spain; 3 Department of Nursing and Physiotherapy University of Cádiz Cádiz Spain; 4 Institute of Research and Innovation in Biomedical Sciences of the Province of Cadiz (INiBICA) Cádiz Spain; 5 Department of Statistics and Operational Research University of Cádiz Cádiz Spain

**Keywords:** chronic pain, mHealth, mobile health, mobile app, health app, digital intervention, monitoring, pain intensity, quality of life, functionality, disability, disabilities, systematic review, review methodology, search strategy, library science, RCT, randomized controlled trial, pain, health outcome, self-management

## Abstract

**Background:**

Chronic pain (CP) is 1 of the leading causes of disability worldwide and represents a significant burden on individual, social, and economic aspects. Potential tools, such as mobile health (mHealth) systems, are emerging for the self-management of patients with CP.

**Objective:**

A systematic review was conducted to analyze the effects of mHealth interventions on CP management, based on pain intensity, quality of life (QoL), and functional disability assessment, compared to conventional treatment or nonintervention.

**Methods:**

PRISMA (Preferred Reporting Items for Systematic Reviews and Meta-Analysis) guidelines were followed to conduct a systematic review of randomized controlled trials (RCTs) published in PubMed, Web of Science, Scopus, and Physiotherapy Evidence Database (PEDro) databases from February to March 2022. No filters were used. The eligibility criteria were RCTs of adults (≥18 years old) with CP, intervened with mHealth systems based on mobile apps for monitoring pain and health-related outcomes, for pain and behavioral self-management, and for performing therapeutic approaches, compared to conventional treatments (physical, occupational, and psychological therapies; usual medical care; and education) or nonintervention, reporting pain intensity, QoL, and functional disability. The methodological quality and risk of bias (RoB) were assessed using the Checklist for Measuring Quality, the Oxford Centre for Evidence-Based Medicine Levels of Evidence, and the Cochrane RoB 2.0 tool.

**Results:**

In total, 22 RCTs, involving 2641 patients with different CP conditions listed in the *International Classification of Diseases 11th Revision* (ICD-11), including chronic low back pain (CLBP), chronic musculoskeletal pain (CMSP), chronic neck pain (CNP), unspecified CP, chronic pelvic pain (CPP), fibromyalgia (FM), interstitial cystitis/bladder pain syndrome (IC/BPS), irritable bowel syndrome (IBS), and osteoarthritis (OA). A total of 23 mHealth systems were used to conduct a variety of CP self-management strategies, among which monitoring pain and symptoms and home-based exercise programs were the most used. Beneficial effects of the use of mHealth systems in reducing pain intensity (CNP, FM, IC/BPS, and OA), QoL (CLBP, CNP, IBS, and OA), and functional disability (CLBP, CMSP, CNP, and OA) were found. Most of the included studies (18/22, 82%) reported medium methodological quality and were considered as highly recommendable; in addition, 7/22 (32%) studies had a low RoB, 10/22 (45%) had some concerns, and 5/22 (23%) had a high RoB.

**Conclusions:**

The use of mHealth systems indicated positive effects for pain intensity in CNP, FM, IC/BPS, and OA; for QoL in CLBP, CNP, IBS, and OA; and for functional disability in CLBP, CMSP, CNP, and OA. Thus, mHealth seems to be an alternative to improving pain-related outcomes and QoL and could be part of multimodal strategies for CP self-management. High-quality studies are needed to merge the evidence and recommendations of the use of mHealth systems for CP management.

**Trial Registration:**

PROSPERO International Prospective Register of Systematic Reviews CRD42022315808; https://www.crd.york.ac.uk/prospero/display_record.php?RecordID=315808

## Introduction

Chronic pain (CP) is a leading cause of disability worldwide [1], affecting approximately 20% of the global population [2]. Moreover, in developed countries, up to 1 of 5 adults suffers from CP of any type [3]. This condition implies a substantial burden for people, and it also has a social and economic impact on health care systems and employment activity [2]. In fact, although the direct health care costs of managing CP conditions are important, the indirect costs, such as disability compensation and work absenteeism, are higher [4].

CP is defined as pain that persists or recurs for longer than 3 months, including a broad range of pain conditions collected in the *International Classification of Diseases 11th Revision* (ICD-11) [5]. It is a new and pragmatic classification system to apply in primary care and clinical settings for specialized pain management [6]. Current pain management interventions are based on multimodal and biopsychosocial models, which include pain education programs, exercise programs, cognitive and behavioral strategies, relaxation techniques, goal setting strategies, self-monitoring symptoms, and self-tailoring strategies [7-9]. Moreover, emotional distress, functional disability, and sleep disturbances are closely linked to the perception of pain and the pain-related outcomes in patients with CP [10,11]. Therefore, strategies for CP management should address all biopsychosocial aspects of this health condition.

Recently, innovative and potential alternatives to support the self-management of patients with CP have emerged, such as mobile device–based health care, or mobile health (mHealth) [12]. mHealth involves the practice of medicine and public health based on mobile devices to improve and promote health status [13]. According to the target of mHealth systems in CP, they can be grouped into 3 categories [12,14]: (1) education, including general information about pain, symptom identification, and treatment planning; (2) monitoring, tracking daily pain episodes and severity, symptoms, mood, activity, and medication use; and (3) treatment, involving several management strategies. These systems empower patients to become more engaged and encourage self-management [15], improving some pain-related outcomes. In line with this, several pain-related apps have been identified from scientific databases and app stores for the management of a wide range of pain (chronic and acute) conditions [16,17]. Nevertheless, there is a lack of scientific and health professional support in many of the mHealth systems, highlighting the need for developing appropriate apps based on the patient’s requirements, also in the management of CP [18].

The available evidence points out promising effects of internet-delivered interventions on different biopsychosocial aspects of CP. Gandy et al [19] studied the use of these interventions using any type of device and technology for CP, showing small effects on pain intensity and disability outcomes in patients with mixed CP conditions, chronic low back pain (CLBP), fibromyalgia (FM), arthritic conditions, peripheral neuropathy, spinal cord injury, migraine, and chronic pancreatitis. In a similar vein, Moman et al [14] discussed the effects of both electronic health (eHealth), based on web apps, and mHealth technologies in patients with CP (general CP, CLBP, FM, and osteoarthritis [OA]), showing significant improvements in pain intensity outcomes at short-term follow-up. Nevertheless, the study was mainly based on eHealth systems, and few findings were obtained from mobile apps. Du et al [20] analyzed the use of web-health-based interventions and mHealth interventions in patients with CLBP, showing better effects on both pain and disability outcomes in favor of mHealth systems. According to the effects of mHealth, a recent review [21] evaluated the effectiveness of app-based interventions on several CP conditions (general CP, CLBP, chronic neck pain (CNP), rheumatoid arthritis, OA, menstrual pain, frozen shoulder pain, and migraine), stating that these apps are significantly more effective, with a small effect size in reducing pain in comparison to control groups. Thurnheer et al [22] analyzed the efficacy of app usage in the management of patients with cancer and noncancer pain (chronic cancer pain, general CP, CLBP, CNP, menstrual pain, and acute pain), reporting beneficial effects on pain, particularly in an out-clinic setting. The evidence of the use of mHealth systems is still emerging and focusing mainly on its effects on pain intensity. Moreover, commonly studied pain conditions (cancer and noncancer pain) and different types of pain (acute and chronic) are mixed, leading to heterogeneity in their findings.

In view of this background and to the best of our knowledge, none of the published reviews has examined the effects of the use of mHealth systems on pain intensity along with the effects on the functional disability and quality of life (QoL) of patients with CP. Therefore, the main purpose of this systematic review is to determine the effects of the use of mHealth systems on different CP conditions listed in the ICD-11, based on the improvement of pain intensity, QoL, and functional disability, according to the findings reported with randomized controlled trials (RCTs). Furthermore, we provide an overview of the available mHealth systems for CP management, their purposes, and their features.

## Methods

### Study Design

The protocol of this systematic review was registered on the International Prospective Register of Systematic Reviews (PROSPERO) database (CRD42022315808) [23]. It was conducted following the 2020 PRISMA (Preferred Reporting Items for Systematic Reviews and Meta-Analyses) guidelines for systematic reviews of RCTs [24].

### Search Strategy

The search strategy was based on CP diseases according to the ICD-11 [25]. The search was conducted from February to March 2022 in the following databases: PubMed, Web of Science, Scopus, and Physiotherapy Evidence Database (PEDro). The search strategy was first developed for the PubMed database using Medical Subject Headings, and it was adapted for other databases. The search was not filtered either by language or by date of publication. The search strategy for each database is provided in Multimedia Appendix 1.

### Eligibility Criteria

The eligibility criteria were defined according to the PICOS (Population, Intervention, Comparison, Outcomes, Study type) framework [26]. The population included adults (≥18 years old) with any CP condition listed in the ICD-11 [25]. Interventions were mHealth systems based on mobile apps (smartphone or tablet) used for monitoring pain and health-related outcomes, for pain and behavioral self-management, and for performing therapeutic approaches. The rationale for including monitoring apps as an intervention was their effects on modifying the user’s behavior, expectation, and performance for disease management or health promotion [27]. Some of the apps’ features for promoting behavior changes are reminders and notifications, tracking activity, goal planning, and tailored information [28]. For comparison, the control group included conventional treatments (physical, occupational, and psychological therapies; care medical; and education) or nonintervention. Primary outcomes were based on pain intensity, QoL, and functional disability, and only RCTs were included as study designs.

Studies with a sample of children or adolescents; including a pain condition with a duration less than 3 months; based on the management of cancer-related pain or pre- and postsurgery trauma interventions (eg, knee arthroplasty, carpal tunnel syndrome); including websites, text messages, or other devices (eg, smartwatches, laptops); and those in which all studied groups used the mHealth system for the intervention were excluded.

### Study Selection Process

After retrieving the documents from different databases, duplicated documents were removed using Rayyan QCRI (Qatar Computing Research Institute) [29] and manual screening. Studies were first screened by title and abstract by 2 researchers (authors MML and JAMM) according to the eligibility criteria. Next, the full text of potentially relevant papers was reviewed by MML and JAMM to decide whether they should be included in the analysis. Disagreements were discussed and resolved by consensus with a third researcher (author IF).

### Data Extraction

The following data were extracted from the included studies: author, year of publication, and country; CP conditions; total number of participants; demographic information, including age and gender, for each study group; intervention details (type, follow-up assessments, and total study duration); and primary and secondary outcomes, as well as outcome measurements or tools. Furthermore, data of the main findings related to pain intensity, QoL, and functional disability were collected. Finally, specific information about the purpose and main features of the mHealth systems used as interventions was identified.

### Risk of Bias, Methodological Quality, and Level of Evidence Assessment

First, the risk of bias (RoB) was assessed using the Cochrane RoB 2.0 tool [30], including 5 domains and an overall judgment. The 5 domains are (1) bias arising from the randomization process, (2) bias due to deviations from intended interventions, (3) bias due to missing outcome data, (4) bias in measurement of the outcome, and (5) bias in selection of the reported result. Each domain was categorized as “low risk,” “high risk,” or “unclear risk” based on the answers to signaling questions. An overall RoB assessment of the RCTs was performed following the recommendations in the guidance document.

Second, the Checklist for Measuring Quality [31] was used. It includes 26 items categorized by 5 subscales: reporting (9 items), external validity (3 items), bias (7 items), confounding (6 items), and power (1 item). Each item is scored 0 or 1, except for 1 item in the reporting subscale whose score ranges from 0 to 2 and the single item in the power subscale whose score ranges from 0 to 5, with a maximum overall score of 31. A score less than 50% indicates low methodological quality, 50%-65% indicates medium methodological quality, and >65% indicates high methodological quality.

Finally, the levels of evidence were reported according to the 2011 Oxford Centre for Evidence-Based Medicine (OCEBM), concerning the subject area or clinical setting and the study design involving the clinical question [32]. The level of evidence ranged from 1 (strong evidence) to 5 (weak evidence).

These assessments were performed by 2 authors (MML and JAMM), and the discrepancies were solved by agreement with a third researcher (author AS). These discrepancies appeared mainly in the RoB assessment, specifically in some questions related to deviations from intended interventions and measurement of outcomes. We also discussed some items of the Checklist for Measuring Quality corresponding to external (source population) and internal (blinding and concealment) validity and the power effect.

## Results

### Study Selection

**Figure 1:**
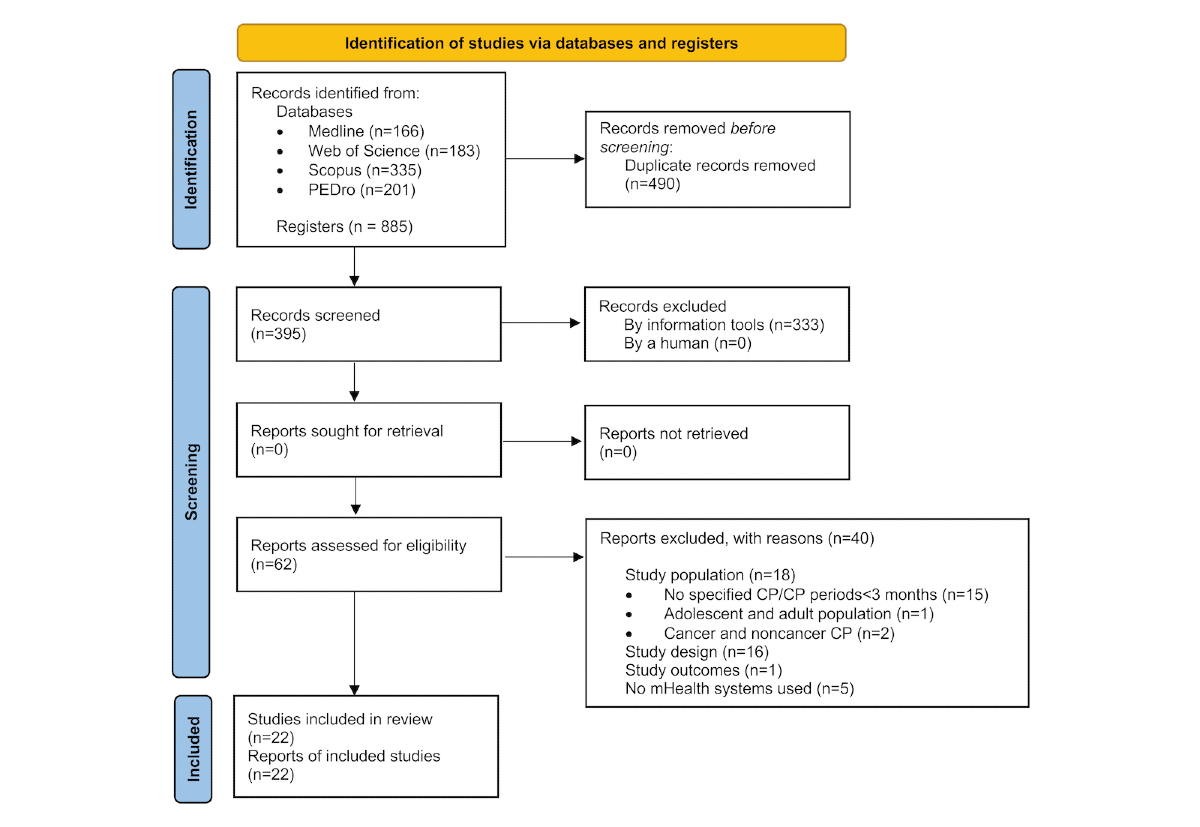
Information flow diagram of the selection process of the systematic review. CP: chronic pain; mHealth: mobile health; PEDro: Physiotherapy Evidence Database.

A total of 885 studies were retrieved from the systematic literature review, of which 490 (55.4%) were duplicates and so deleted automatically. After the first screening by title and abstract, 62/395 (15.7%) studies were selected for full-text reviewing. According to the pre-established selection criteria, a total of 22 (35.5%) studies were finally included in the qualitative analysis. The full screening process and the main reasons for exclusion are shown in Figure 1.

### Risk of Bias, Methodological Quality, and Level of Evidence

Regarding the results of RoB assessment by domain, 18/22 (82%) studies had a low RoB for the random allocation domain and 16/22 (73%) studies had a RoB for the missing outcome data domain. For the second (bias due to deviations from intended interventions) and fourth (measurement of outcomes) domains, 11 (50%) and 14 (64%) studies had some concerns, respectively. Last, in the selection of the reported results domain, 16 (73%) studies had a low RoB but 3 (14%) studies had a high RoB. For overall judgment, 7/22 (32%) studies had a low RoB for their outcomes, 10/22 (45%) studies had some concerns, and 5/22 (23%) studies had a high RoB.

Regarding the Checklist for Measuring Quality, 18 (82%) studies [33-50] reported medium methodological quality (between 50% and 65%), and the rest [51-54] scored high on methodological quality (>65%). Based on the clinical settings of the included studies, which concern therapy or treatment, the OCEBM level of evidence was based on systematic reviews of RCTs or, failing that, individual RCTs with narrow 95% CIs. Thus, all included papers yielded an OCEBM level of 2 for a clinical question of treatment benefits, considering them as highly recommendable.

**Figure 2:**
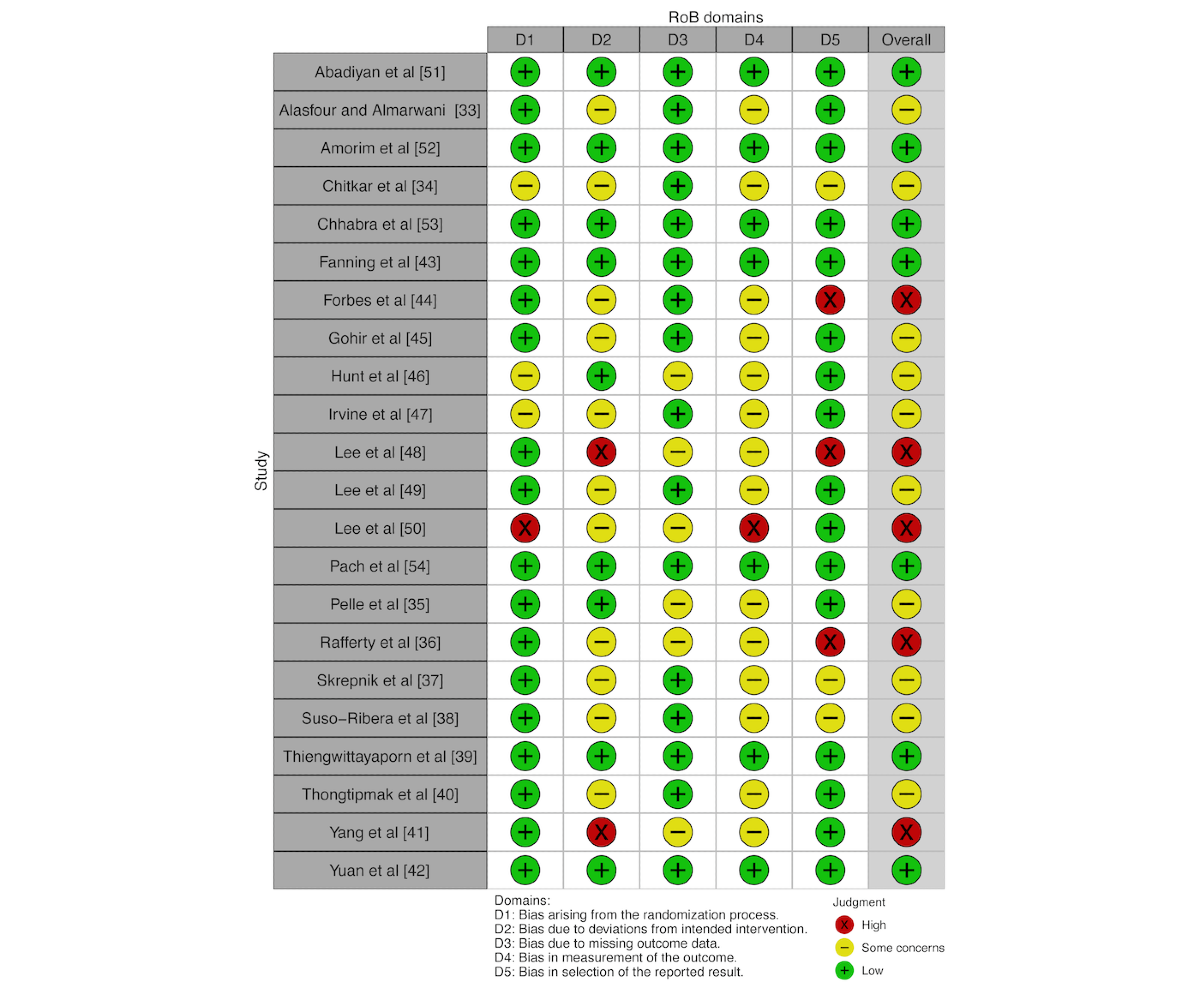
RoB assessment: traffic light plot. RoB: risk of bias.

**Figure 3:**
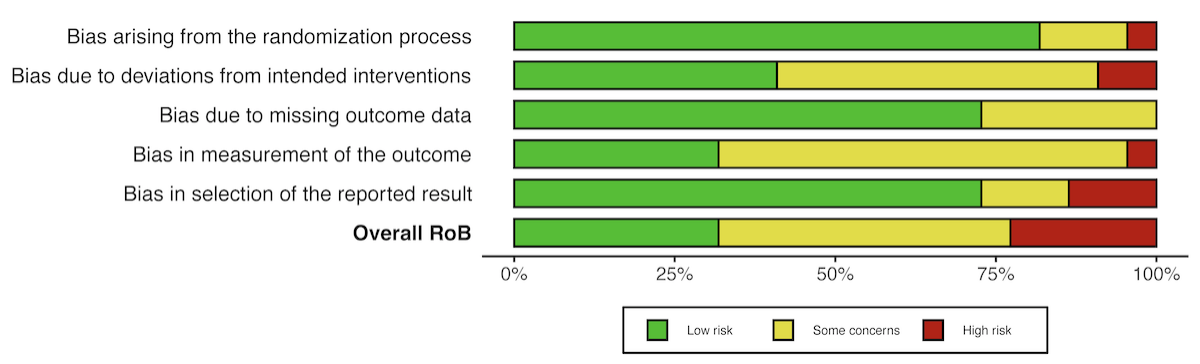
RoB assessment: summary plot. RoB: risk of bias.

Detailed results of the RoB assessment are shown in Figures 2 and 3. The methodological quality and the level of evidence and degrees of recommendation of the included studies are detailed in Multimedia Appendix 2 [33-54] .

### Study Characteristics

The main characteristics of the studies included are shown in Table 1. Publication dates ranged from 2015 to 2022. A total of 2641 patients with CP were involved in this present systematic review, 70.6% (1793/2539) being female. The average age was 38.93 (SD 59.29) years, excluding 1 (5%) study [47] in which this information was not available.

According to CP conditions listed in the ICD-11, OA is the condition most studied in the literature, followed by CLBP [41,47,52,53] and CNP [40,49,51,54]. The lowest studies were chronic pelvic pain (CPP) [44] and interstitial cystitis/bladder pain syndrome (IC/BPS) [50].

**Table 1:** Study characteristics.

Characteristics			Value
**Year of publication (N=22), n (%)**			
	2015-2018	6 (27.3)	
	2019-2022	16 (72.7)	
**Region where the study took place (N=22), n (%)**			
	Asia	11 (50.0)	
	Europe	4 (18.2)	
	North America	5 (22.7)	
	South America	1 (4.5)	
	Oceania	1 (4.5)	
Age (years)^a^, mean (SD)			38.93 (59.29)
**Gender (N=2539)^b^, n (%)**			
	Female	1793 (70.6)	
	Male	746 (29.4)	
**CP^c^ conditions (N=22), n (%) **			
	CLBP^d^ [41,47,52,53]	4 (18.2)	
	CMSP^e^ [38]	1 (4.5)	
	CNP^f^ [40,49,51,54]	4 (18.2)	
	CP (unspecified) [43]	1 (4.5)	
	CPP^g^ [44]	1 (4.5)	
	FM^h^ [42,48]	2 (9.1)	
	IC/BPS^i^ ]50]	1 (4.5)	
	IBS^j^ [36,46]	2 (9.1)	
	OA^k^ [33-35,37,39,45]	6 (27.3)	
**Interventions based on mHealth^l^ systems (N=22), n (%)**			
	Home-based PA^m^ program	9 (40.9)	
	Education	8 (36.4)	
	CBT^n^	4 (18.2)	
	Monitoring pain-related outcomes and symptoms	10 (45.5)	
	Monitoring PA parameters	11 (50.0)	
	Mind relaxation techniques	5 (22.7)	
**Intervention period (N=22), n (%)**			
	<3 months	15 (68.2)	
	3-6 months	7 (31.8)	
**Outcomes assessed (N=22), n (%)**			
	Pain intensity	17 (77.3)	
	QoL^o^	15 (68.2)	
	Functional disability	17 (77.3)	

^a^Average age of available data except for 1 study.

^b^Gender proportion of available data except for 1 study.

^c^CP: chronic pain.

^d^CLBP: chronic low back pain.

^e^CMSP: chronic musculoskeletal pain.

^f^CNP: chronic neck pain.

^g^CPP: chronic pelvic pain.

^h^FM: fibromyalgia.

^i^IC/BPS: interstitial cystitis/bladder pain syndrome.

^j^IBS: irritable bowel syndrome.

^k^OA: osteoarthritis.

^l^mHealth: mobile health.

^m^PA: physical activity.

^n^CBT: cognitive behavioral therapy.

^o^QoL: quality of life.

### Types of mHealth and Comparison Interventions

Several approaches for the self-management of patients with CP involved mHealth systems. On the one hand, we found the monitorization of pain-related variables and symptoms as part of the interventions, either isolated [37,38,41,48] or in combination with other management strategies [39,40,42,47,49,50]. Similarly, the tracking of physical activity (PA) parameters (daily PA and mobility, PA-related goals achieved, and adherence) was also used in 11 (50%) studies [33,35,37,40,41,43,45,49,51-53] aiming to record PA-related goals and to enhance PA performance and behaviors. On the other hand, self-management of CP focused on home-based PA programs as the most common intervention [33,35,39,40,45,49,51,53,55], including a wide variety of exercises, both general and specific for this population. Other common self-management approaches were educational sessions and materials [34-36,45-47,50,55]. Less frequent strategies were cognitive behavioral therapy (CBT) [43,46,47,54] and relaxation and mind-body techniques [43,44,46,54,55]. A total of 23 mHealth systems were used for monitoring [37,38,41,48,52], treatment strategies [34,36,44,46,54], and a combination of both [33,35,39,40,42,43,45,47,49-51,53]. Detailed information about the mHealth systems, their purpose of use, and the principal features are summarized in Multimedia Appendix 3.

In the control groups, interventions were based on usual health care (medical and physical therapies), being the most common comparison intervention [35,37,38,41,44,45,47-54]. Other papers performed the same intervention in both groups, one using mHealth and the other using traditional methods [33,34,36,39,42]. Finally, only 3 (14%) studies [40,43,46] did not involve any intervention.

### Study Outcomes and Measurement Tools Used

Pain intensity was assessed in a total of 17 (77%) studies (N=1780). The numeric rating scale (NRS) [33,37,38,45,52-54] and the visual analogue scale (VAS) [40-42,48-51] were the most used. Regarding the OA condition, the Knee injury and Osteoarthritis Outcome Score (KOOS), the Hip injury and Osteoarthritis Outcome Score (HOOS), and the Western Ontario and McMaster (WOMAC) questionnaires were specific tools also used to assess pain intensity [34,35,39].

There was a wide range of tools used in 15 (68%) studies (N=1744) for assessing the QoL. The most repeated instruments were the 36-item Short Form Health Survey (SF-36) [34,41,43,49-51], followed by the EuroQoL-5D [35,48].

In the case of functional disability [33-35,38,39,41-45,47-53], 17 (77%) studies (N=1928) assessed it. Although there are different tools for assessing this outcome, they usually focus on a specific condition (Multimedia Appendix 3). For example, for patients with CNP [49,51], the Neck Disability Index (NDI) was used; for patients with FM, the Fibromyalgia Impact Questionnaire (FIQ) [42,48] was used; and for patients with OA, WOMAC [33,34,45], KOOK [35,39] and HOOS [35] were used.

### Effects of mHealth Interventions vs Control Groups

To provide an overview of the differences found between mHealth interventions and control groups in the included studies, a visual representation is shown in Tables 2-5. The “*” sign indicates significance in favor of the mHealth intervention group, and the “=” sign indicates no significant differences between groups. No significant differences in favor of the control groups were reported.

Results of home-based PA programs delivered by mHealth systems led to a significant improvement in pain intensity in patients with CNP [49,51] and OA [35,45] when compared to usual care. Likewise, this type of intervention had significant effects on functional disability [35,45,49,51,53], but only Abadiyan et al [51] showed significant differences in the QoL between groups. In addition, when home-based PA programs delivered by mHealth systems were compared with similar traditional methods, a significant improvement in favor of mHealth for pain intensity [33], QoL, and functional disability outcomes [39] was observed in patients with OA and CNP. Nevertheless, no significant differences were obtained for any of the outcomes measured in patients with FM [42].

In relation to educational interventions based on mHealth, improvements in the QoL in OA [34], IBS [36], and IC/BPS [50] conditions were observed when compared either to usual care or to similar intervention by traditional methods. This intervention also showed improvements in functionality and pain intensity in patients with OA [34] but not for pain intensity in patients with IC/BPS [50].

CBT based on mHealth systems showed some significant improvements in QoL [46,47] and functional disability [47] in favor of the mHealth group when compared to usual care or no intervention. Nevertheless, this intervention neither reduced pain intensity in CNP [54] nor improved the QoL and functional disability in CP significantly [43].

Finally, the results of mHealth interventions focused on monitoring pain and symptoms, compared to usual care, were inconclusive. Thus, significant improvements in reducing pain were reported for patients with OA [37] and FM [48] but not for those with CMSP [38] and CLBP [41]. In patients with CMSP and CLBP, functional disability outcomes significantly improved in favor of mHealth groups [38,41], while those diagnosed with FM did not achieve significant improvements in this outcome [48]. No significant changes in the overall QoL were observed between groups with this type of intervention [41,48].

Other interventions, such as isolated monitoring of PA parameters [52] and mindfulness meditation alone [44], did not show significant differences between the mHealth and control groups for any of the studied outcomes.

With regard to the reporting of adverse events or treatment reactions of the studied interventions, only 6 (27%) of the 22 studies [37,38,45,50,51,54] provided this information, of which only 1 (17%) [54] recorded serious adverse events (cancer, sudden hearing loss, nerve injury and spinal tap, tonsillectomy, and accident causing a fracture), but none of them was considered related to the trial intervention.

**Table 2:** Characteristics of participants and study interventions (studies 1-11).

Study	CP^a^ condition	Participants, N, intervention group (IG), n (%), control group (CG), n (%); age (years), mean (SD); gender (% female)	Intervention		Total study duration (weeks); follow-up period
			mHealth^b^	Control	
Alasfour and Almarwani [33]	Knee OA^c^	N=40; 54.40 (4.33); 100% IG: n=20; 53.65 (3.96); 100% CG: n=20; 55.15 (4.64); 100%	Home-based PA^d^ program (lower-limb-strengthening exercises) with the My Dear Knee app; also, exercise adherence and completed sessions recorded by the app	Home-based PA program through paper handouts	6; 3rd and 6th weeks
Arfaei Chitkar et al [34]	Knee OA	N=60; 58.17 (7.55); 100% IG: n=31; 57.84 (8.63); 100% CG: n=29; 58.52 (6.33); 100%	Educational content through the mobile app; usual medical care	Educational content without the app; usual medical care	8; 2nd month
Pelle [35]	Knee or hip OA	N=427 IG: n=214; 62.1 (7.7); 68.7% CG: n=213; 62.1 (7.0); 74.7%	Home-based PA program and education content provided by the Dr. Bart app, with also PA-related goals, self-monitoring, and motivational reminders	Usual care with no active treatment	24; 3rd and 6th months
Rafferty et al [36]	IBS^e^	N=25 IG: n=14; 27.2 (9.5); 86% CG: n=11; 25.7 (11.9); 91%	Nutrition information and recommendations based on patient-specific and individualized diet plans through the Heali app; standard dietary education materials (online)	Standard dietary education materials (online)	4; 1st month
Skrepnik et al [37]	Knee OA	N=211; 62.6 (9.4); 50.2% IG: n=107; 61.6 (9.5); 55.1% CG: n=104; 63.6 (9.3); 45.2%	Monitoring pain, PA parameters, and mood data with feedback and motivational messages from the OA GO app; standard-of-care instructions and education; unblinded wearable device	Standard-of-care instructions and education; blinded wearable device	12; 1 week, 1st and 3rd months
Suso-Ribera et al [38]	CMSP^f^	N=165; 52.1 (11.2); 73.8% IG-1: 53 IG-2: 56 CG: 56	IG-1: monitoring pain-related outcomes using the Pain Monitor app with alarms and usual care; IG-2: monitoring pain-related outcomes with the Pain Monitor app without alarms and usual care	Usual care	4; 1st month
Thiengwittayaporn et al [39]	Knee OA	N=82 IG: n=42; 62.2 (6.8); 85.7% CG: n=40; 63.0 (9.7); 92.5%	Home-based PA program and education, and disease monitoring (symptoms and stages) with the Rak Kao app	Standard education and exercise instructions through handouts	4; 1st month
Thongtipmak et al [40]	CNP^g^	N=100 IG: n=50; 22.86 (1.99); 82% CG: n=50; 22.68 (2.23); 76%	Home-based PA program and monitoring pain level before and after exercises with the NeckProtector app	Rest	Same day
Yang et al [41]	CLBP^h^	N=8 IG: n=5; 35 (10.93); 20% CG: n=3; 50.33 (9.29); 100%	Monitoring pain intensity and activity levels using the Pain Care app; self-management program based on individualized exercises and physiotherapy treatment	Only physiotherapy treatment	4; 2nd and 4th weeks
Yuan et al [42]	FM^i^	N=40 IG: n=20; 43.3 (8.4); 95% CG: n=20; 42.1 (11.8); 100%	Self-care management based on education, home-based PA, and sleep hygiene and relaxation techniques using the ProFibro app, with also self-monitoring disease impact according to FIQ domains; usual medical care	Traditional paper book of similar content; usual medical care	6; 6th week
Fanning et al [43]	CP	N=28; 70.21 (5.22); 78.6% IG: n=15; 70.12 (5.43); 86.7% CG: n=13; 70.32 (5.20); 69.2%	Monitoring PA-related goals, CBT^j^, and mindfulness-based relapse prevention using the Mobile Health Intervention to Reduce Pain and Improve Health [MORPH] Companion and Fitbit apps	Waitlist	12; 3rd month

^a^CP: chronic pain.

^b^mHealth: mobile health.

^c^OA: osteoarthritis.

^d^PA: physical activity.

^e^IBS: irritable bowel syndrome.

^f^CMSP: chronic musculoskeletal pain.

^g^CNP: chronic neck pain.

^h^CLBP: chronic low back pain.

^i^FM: fibromyalgia.

^j^CBT: cognitive behavioral therapy.

**Table 3:** Characteristics of participants and study interventions (studies 12-22).

Study	CP^a^ condition	Participants, N, intervention group (IG), n (%), control group (CG), n (%); age (years), mean (SD); gender (% female)	Intervention		Total study duration (weeks); follow-up period
			mHealth^b^	Control	
Forbes et al [44]	CPP^c^	N=90 IG-1: n=31; 34.8 (9.9); 100% IG-2: n=30; 35.7 (5.7); 100% CG: n=29; 35.0 (8.6); 100%	IG-1: mindfulness meditation course delivered by the Headspace app and usual care; IG-2: muscle relaxation techniques in the app and usual care	Usual care	8; 2nd, 3rd, and 6th months
Gohir et al [45]	Knee OA^d^	N=105 IG: n=48; 65.2 (9.7); 70.8% CG: n=57; 68.0 (8.6); 64.9%	Home-based PA^e^ program, including strengthening, core stability and balance exercises, and educational sessions, provided by the Hereafter app	Usual care	6; 6th week
Hunt et al [46]	IBS^f^	N=121; 32 (10.2); 75.2% IG: n=62 CG: n=59	Psychoeducation, CBT^g^, relaxation techniques, and information about diet, provided by the Zemedy app	Waitlist	8; 2nd month
Irvine et al [47]	CLBP^h^	N=597 IG-1: n=199; 58.3% IG-2: n=199; 58.8% CG: n=199; 62.8%	IG-1: CBT and education through the FitBack app, with also recording of pain-related outcomes; IG-2: alternative care by emails with internet resources (both groups received weekly reminder prompts and emails for assessments)	Usual care; only contacted for assessments	8; 2nd and 4th months
Lee et al [48]	FM^i^	N=25 IG: n=14; 42.8 (7.2); 100% CG: n=11; 41.7 (11.2); 100%	Monitoring pain-related outcomes (intensity, frequency, and environmental factors) with the Pain Assessment and Analysis System [PAAS] Clinic app	Usual care	12; 1st and 3rd months
Lee et al [49]	CNP^j^	N=20 IG: n=11; 27.09 (4.83); 55% CG: n=9; 27.56 (4.67); 45%	McKenzie neck exercise program with a smartphone app in the workplace environment, with also a self-feedback function and monitoring pain	Written instructions about postural hygiene	8; 2nd month
Lee et al [50]	IC/BPS^k^	N=56 IG: n=29; 42.9 (10.4); 100% CG:n=27; 46.3 (14.2); 100%	Health education and symptom self-management with the Taiwan Interstitial Cystitis Association [TICA] app; patients could continue using usual care	Usual care	8; 2nd month
Abadiyan et al [51]	CNP	N=60; 38.5 (9.1) IG-1: n=20; 41.3 (8.1); 50% IG-2: n=20; 40.3 (7.9); 50% CG: n=20; 37.4 (9.8); 35%	IG-1: home-based PA program, global posture re-education (GPR), and self-managed work time with the Seeb app, with also recording of PA parameters; IG-2: GPR alone	Traditional neck education and exercise therapy	8; 8th week
Amorim et al [52]	CLBP	N=68 IG: n=34; 59.5 (11.9); 44% CG: n=34; 57.1 (14.9); 56%	Monitoring PA-related goals with the IMPACT app, with motivational messages; telephone-based coaching sessions; PA and sedentary behavior information booklet	PA information booklet and advice to stay active	24; weekly and 6th month
Chhabra et al [53]	CLBP	N=93 IG: n=45; 41.4 (14.2) CG: n=48; 41.0 (14.2)	Home-based PA program, including specific back exercises and aerobic PA; monitoring daily PA parameters with the Snapcare app; written prescription and usual medical care	Written prescription, including PA advice; usual medical care	12; 3rd month
Pach et al [54]	CNP	N=220 IG: n=110; 37.9 (11); 67.3% CG: n=110; 39.8 (11.6); 71.8%	Relaxation exercises (autogenic training, mindfulness meditation, and guided imagery) and CBT strategies with the RelaxNeck app; follow-up data collected using app-based questionnaires	Usual care; app for data entry only	24; 3rd and 12th months

^a^CP: chronic pain.

^b^mHealth: mobile health.

^c^CPP: chronic pelvic pain.

^d^OA: osteoarthritis.

^e^PA: physical activity.

^f^IBS: irritable bowel syndrome.

^g^CBT: cognitive behavioral therapy.

^h^CLBP: chronic low back pain.

^i^FM: fibromyalgia.

^j^CNP: chronic neck pain.

^k^IC/BPS: interstitial cystitis/bladder pain syndrome.

**Table 4:** Overall RoB^a^ assessment, study outcomes, and main results (studies 1-11).

Study	CP^b^ condition	Study outcomes (measurement tools)			RoB		Outcome results^c^		
		Primary	Secondary			Pain intensity		QoL^d^	Functional disability
Alasfour and Almarwani [33]	Knee OA^e^	Self-reported exercise adherence (percentage of completed exercises)	Pain intensity (Arabic version of the numeric pain rating scales [ANPRS]) Physical function (ArWOMAC^f^) Lower-limb muscle strength (Five-Times Sit-to-Stand Test [FTSST])	–		*		N/A^g^	**=**
Arfaei Chitkar et al [34]	Knee OA	Physical functioning (WOMAC^h^) QoL (SF-36^i^)	N/A	–		*		*	*
Pelle [35]	Knee/hip OA	Number of self-reported consultations in health care	Pain intensity, symptoms, and functional limitations (KOOS^j^/HOOS^k^) QoL (EuroQoL-5D-3L) PA^l^ level (Short Questionnaire to Assess Health-enhancing physical activity [SQUASH]) Patient’s cognitive and emotional perceptions (brief Illness Perception Questionnaire [IPQ]) Knowledge, skills, and confidence (PAM-13)	–		*		=	*
Rafferty et al [36]	IBS^m^	IBS symptoms (5-item IBS Symptom Severity Scale [IBSS-SSS]; Rome IV) QoL (World Health Organization Quality of Life [WHOQOL-BREF]) LFD knowledge (low FODMAP dietary consumption questionnaire [LFDA Quest.]) LFD adherence (low FODMAP dietary knowledge questionnaire [LFDK Quest.])	N/A	X		N/A		*	N/A
Skrepnik et al [37]	Knee OA	Mobility (6-minute walking test [6MWT]; steps/day)	Pain intensity (NRS^n^) Patient and physical satisfaction (PAM-13) Quality of sleep (wearable activity monitor) Mood states (visual analogue mood scale [VAMS]) Treatment-emergent adverse events (treatment-emergent adverse events [TEAEs])	–		*		N/A	N/A
Suso-Ribera et al [38]	CMSP^o^	Pain intensity (NRS) Medication side effects (NRS)	Pain-related interference (NRS) Fatigue (NRS) Depression, anxiety, and anger (NRS)	–		=		N/A	*
Thiengwittayaporn et al [39]	Knee OA	Patient’s ability to correctly perform the exercises (80% completed exercise repetitions)	Range of motion (goniometer) Pain intensity, symptoms, daily life activities, PA and sports performed, and QoL (KOOS) Satisfaction/expectation with functional ability (Knee Society Score [KSS])	+		=		*	*
Thongtipmak et al [40]	CNP^p^	Pain intensity (VAS^q^) Muscle tension (VAS) Pressure pain threshold (pressure algometry) Cervical range of motion (CROM; device) Acceptability assessment (System Usability Scale [SUS])	N/A	–		*		N/A	N/A
Yang et al [41]	CLBP^r^	Pain intensity (VAS) Disability (Roland-Morris Disability Questionnaire [RMDQ]) QoL (SF-36) Self-efficacy (Pain Self-Efficacy Questionnaire [PSEQ])	N/A	X		=		=	*
Yuan et al [42]	FM^s^	QoL (FIQ^t^)	Pain intensity (VAS) Function (FIQ-Function) Painful body regions (Widespread Pain Index [WPI]) Symptom Severity (SS) scale Self-care (Appraisal of Self-Care Agency Scaled-Revised [ASAS-R])	+		=		=	=
Fanning et al [43]	CP	QoL (SF-36) Physical functioning (SF-36: physical functioning subscale) Self-efficacy for walking (8-item scale) Satisfaction with physical functioning (7-item scale)	N/A	+		N/A		**=**	**=**

^a^RoB: risk of bias; interpretation of RoB: +, low RoB; –, some concerns; X, high RoB.

^b^CP: chronic pain.

^c^Interpretation of outcome results: *, significant differences (*P*<.05) in favor of the mHealth group; =, nonsignificant differences between groups.

^d^QoL: quality of life.

^e^OA: osteoarthritis.

^f^ArWOMAC: Arabic version of Western Ontario and McMaster.

^g^N/A: not applicable.

^h^WOMAC: Western Ontario and McMaster.

^i^SF-36: 36-item Short-Form Health Survey.

^j^KOOS: Knee injury and Osteoarthritis Outcome Score.

^k^HOOS: Hip injury and Osteoarthritis Outcome Score.

^l^PA: physical activity.

^m^IBS: irritable bowel syndrome.

^n^NRS: numeric rating scale.

^o^CMSP: chronic musculoskeletal pain.

^p^CNP: chronic neck pain.

^q^VAS: visual analogue scale.

^r^CLBP: chronic low back pain.

^s^FM: fibromyalgia.

^t^FIQ: Fibromyalgia Impact Questionnaire.

**Table 5:** Overall RoB^a^ assessment, study outcomes, and main results (studies 12-22).

Study	CP^b^ condition	Study outcomes (measurement tools)			RoB		Outcome results^c^		
		Primary	Secondary			Pain intensity		QoL^d^	Functional disability
Forbes et al [44]	CPP^e^	Pain-related disability (Chronic Pain Grade-Disability subscale) QoL (RAND 36) Pain acceptance (chronic pain acceptance questionnaire [CPAQ]) Depression and anxiety (HAD) Self-efficacy (Pain Self-efficacy Quest.) Sexual health (sexual health outcomes in women questionnaire [SHOW-Q]) Mindfulness (Cognitive and mindfulness-revised scale) Individualized outcome (Measure yourself medical outcome profile [MYMOP])	Study feasibility (CPAQ) App usability (System Usability Scale [SUS]) Adherence to the app (frequency of app use)	X		N/A^f^		**=**	**=**
Gohir et al [45]	Knee OA^g^	Pain intensity (NRS^h^)	Physical functioning (WOMAC^i^, Timed Up & Go [TUG], and 30-second sit-to-stand test) QoL (Musculoskeletal Health Questionnaire [MSK-HQ]) Symptoms sensory (pressure pain threshold [PPT])	–		*		**=**	*
Hunt et al [46]	IBS^j^	QoL (Irritable Bowel Syndrome Quality of Life [IBS-QOL]) Symptom severity (Gastrointestinal Symptom Rating Scale-IBS [GSRS-IBS])	Diagnostic criteria for IBS (Rome IV) Fear of food (Fear of Food Questionnaire [FFQ]) Gastrointestinal (GI) symptom–specific anxiety (Visceral Sensitivity Index [VSI]) Cognitions-related impact (Gastrointestinal Cognition Questionnaire [GI-COG]) Depression and anxiety (Depression Anxiety Stress Scale [DASS]) Diagnosis and depressive symptom severity (Patient Health Questionnaire [PHQ]) Dose (number of app modules completed)	–		N/A		*	N/A
Irvine et al [47]	CLBP^k^	Physical outcomes: Pain intensity, episodes, and duration (back pain scales) Daily pain management activities Functionality (10-item scale based on Multidimensional Pain Inventory Interference Scale [MPI] and Brief Pain Inventory [BPI]) QoL (Dartmouth Primary Care Cooperative Information Project [Dartmouth CO-OP] scale)	Prevention-helping behaviors Worksite outcomes: Worker productivity (4-item Work Limitations Questionnaire [WLQ]) Presenteeism (Stanford Presenteeism Scale) Other outcomes: Responsibility of own health (Patient Activation Measure [PAM]) Behavior constructs (knowledge, behavioral intentions, and self-efficacy) Attitudes toward pain (10-item Survey of Pain Attitudes [SOPA]) Catastrophizing of pain (Tampa scale)	–		N/A		*	*
Lee et al [48]	FM^l^	Pain intensity (VAS^m^)	QoL (EuroQoL-5D) Disease impact (FIQ^n^) Depression index (Beck’s Depression Index [BDI]) Patient global assessment (patient global assessment [PtGA])	X		*		**=**	**=**
Lee et al [49]	CNP^o^	Pain intensity (VAS) Functional disability (NDI^p^)	QoL (SF-36^q^) Maximal voluntary strength (digital handheld dynamometer) Fear avoidance belief (Fear-Avoidance Belief Questionnaire [FABQ]) Exercise adherence (app)	–		*		=	*
Lee et al [50]	IC/BPS^r^	QoL (SF-36)	Pain intensity (VAS) Symptoms (O´Leary-Sant symptom) Physical function, role physical, bodily pain, vitality, social function, role emotional and mental health (SF-36 subscales)	X		*		=	=
Abadiyan et al [51]	CNP	Pain intensity (VAS)	Disability (NDI) QoL (SF-36) Endurance (progressive isoinertial lifting evaluation [PILE] test) Forward head posture (craniovertebral angle)	+		*		*	*
Amorim et al [52]	CLBP	Pain intensity (NRS) Disability (Roland-Morris Disability Questionnaire [RMDQ]) Care seeking (health care consultations)	Self-reported PA^s^ level (International Physical Activity Questionnaire [IPAQ]) PA data (accelerometer)	+		=		N/A	**=**
Chhabra et al [53]	CLBP	Pain intensity (numeric pain rating scales [NPRS]) Disability (Modified Oswestry Disability Index [MODI])	Only for GI: Daily PA (activity tracker built within the app) Progress in symptoms (Current Symptom Score [CSS])	+		=		N/A	*
Pach et al [54]	CNP	Pain intensity during first 3 months (NRS)	Pain intensity, weekly and during the 6 months (NRS) Pain acceptance (CPAQ) Neck pain–related stress Sick leave days Pain medication intake Adherence	+		=		N/A	N/A

^a^RoB: risk of bias; interpretation of RoB: +, low RoB; –, some concerns; X, high RoB.

^b^CP: chronic pain.

^c^Interpretation of outcome results: *, significant differences (*P*<.05) in favor of the mHealth group; =, nonsignificant differences between groups.

^d^QoL: quality of life.

^e^CPP: chronic pelvic pain.

^f^N/A: not applicable.

^g^OA: osteoarthritis.

^h^NRS: numeric rating scale.

^i^WOMAC: Western Ontario and McMaster.

^j^IBS: irritable bowel syndrome.

^k^CLBP: chronic low back pain.

^l^FM: fibromyalgia.

^m^VAS: visual analogue scale.

^n^FIQ: Fibromyalgia Impact Questionnaire.

^o^CNP: chronic neck pain.

^p^NDI: Neck Disability Index.

^q^SF-36: 36-item Short-Form Health Survey.

^r^IC/BPS: interstitial cystitis/bladder pain syndrome.

^s^PA: physical activity.

## Discussion

### Principal Findings

This study provided an overview of the use of mHealth systems for the self-management of patients with different CP conditions. To the best of our knowledge, this is the first systematic review that identifies the available mHealth interventions and their effects on pain intensity, QoL, and functional disability in patients with CP. Results showed that some interventions based on mHealth systems have beneficial effects on reducing pain and functional disability and improving the QoL. Thus, the scientific evidence suggests that these systems could be a promising alternative in CP self-management through multimodal approaches.

Regarding the analyzed outcomes, 9 of the 17 studies assessing pain intensity [33-35,37,40,45,48,49,51] showed significant effects in reducing pain in favor of mHealth groups. There are several systematic reviews and meta-analyses that support these findings. Pfeifer et al [21] showed that mHealth apps are more effective in reducing pain when compared to control interventions in patients with different CP conditions, such as general CP, CLBP, CNP, arthritis (rheumatoid and OA), menstrual pain, frozen shoulder pain, and migraine. Nevertheless, the authors stated that most of the included studies used cointerventions (eg, physiotherapy, self-management booklets, pharmacological approach, and wearable activity monitors), in addition to using mHealth systems. Likewise, Moman et al [14] observed significant short- and intermediate-term improvements in pain-related outcomes in patients with CP, CLBP, FM and OA, and Thurnheer et al [22] reported a decrease in pain severity in patients with several CP diagnoses (chronic cancer pain, general CP, CLBP, CNP, menstrual pain, and also acute pain) using mobile apps for their management. Furthermore, focusing on the CP condition, Du et al [20] indicated that mHealth-based self-management programs for reducing pain show clinically important effects. Similarly, Chen et al [56] showed that the use of mobile apps for delivering PA programs is associated with significant pain relief in patients with knee OA or chronic knee pain.

Regarding the QoL, improvements were observed in 7 of 15 studies [34,36,39,46,47,50,51] involving several CP conditions (OA, CNP, CLBP, IBS, and IC/BPS). This result agrees with a previous systematic review [22] reporting that patients using a mHealth app for their self-management have a higher QoL compared to patients not using that system. Nevertheless, in the meta-analysis carried out by Chen et al [56], when analyzing the type of technology used for delivering PA programs, they observed that the use of the web is associated with significant improvements in the QoL in patients with knee OA or chronic knee pain, but the use of mobile apps or smartphones is not. This may be because only few of the studies included in this meta-analysis used mobile apps to deliver the interventions, making it difficult to examine the effects of this type of technology.

In the case of functional disability, we found some significant differences between mHealth and control groups in patients with musculoskeletal pain (CLBP [41,47,53], CNP [49,51], and CMSP [38]) and OA [34,35,45]. Nevertheless, these findings are not in line with the available literature. Chen et al [56] did not find evidence for a significant improvement in physical function with technology-supported PA programs. Likewise, results of meta-analyses of telehealth-based interventions, including mHealth and eHealth systems, have suggested that these interventions have no significant effects on physical functionality [14] and disability [57] at short- and intermediate-term follow-up. However, these results are provided by different technology-based interventions and not specifically mHealth systems, which are more recent technologies not sufficiently researched yet.

The types of intervention of the studies included in this systematic review were home-based PA programs, education, CBT, mind-body therapies, and monitoring. This is in line with a large review of the recommendations from clinical practice guidelines (CPG) for musculoskeletal pain, where 3 pillar interventions were identified as key self-management approaches: education, PA, and psychosocial therapies [58]. Similarly, Geraghty et al [59] analyzed the available self-management interventions for chronic widespread pain, with PA programs and medical information being the 2 most common components, followed by psychological approaches. Our findings reported that depending on the type of interventions carried out by mHealth, there are differences in their effects on study outcomes. In this regard, home-based PA programs and education, combined or isolated, showed significant effects on all outcomes compared to other interventions, especially in the case of functional disability. We also found that PA programs and education are commonly considered as cointerventions.

The use of PA as a clinical intervention is suggested as being adequate for several of the conditions included in this systematic review. In patients with OA, it showed a moderate effect on physical functioning, with high patient acceptability and limited side effects, being strongly recommended as conservative management [60]. Similarly, van Doormaal et al [61] reported that PA reduces pain and improves physical function and QoL, with strong-to-moderate evidence. Finally, the CPG for OA include specific exercise programs as core treatment of the nonsurgical management of this condition [58,62]. Moreover, for CLBP and CNP self-management, PA showed significant improvements in pain intensity and functional disability outcomes and slightly more effects on the QoL. In line with this, Bertozzi et al [63] and Price et al [64] reported significant improvement effects of PA programs on CNP in the short and intermediate terms. Nevertheless, both studies have mentioned that the effects of PA are not maintained in the long term, although no high-quality trials are available [63,64]. In the case of FM, although only Yuan et al [42] performed a home-based PA program, this type of intervention is strongly recommended in clinical guidelines for the management of this pathology [65,66]. In fact, previous evidence supports the effectiveness of different modalities of exercise (aerobic, strength, and functional training) in common symptoms of FM and QoL [67].

Education is also considered an essential component of conservative management. In fact, the included studies on several CP conditions applied this approach in isolation or in combination with other interventions, showing improvements in pain-related outcomes, functional disability, and QoL. Education usually includes information about the condition, its prognosis, possible consequences, associated factors, the importance of maintaining a healthy lifestyle, and self-care management options [58,60]. Education promotes feelings of hope and optimism and a positive expectation of the treatment benefits in patients with CP [62].

As previously mentioned, another key purpose of the CPG was to address the psychosocial factors related to CP, for which the internet-delivered interventions may be 1 means of increasing remote access to psychological care. In fact, the previous literature shows beneficial effects of internet-delivered cognitive and behavioral interventions for CP on pain intensity, disability, mood states, and QoL, supporting the use of technological devices for pain management outcomes [19,68]. In that line, CBT is the most studied and used and is especially important in some CP conditions, such as FM [65]. Evidence showed that patients with FM who received CBT showed reduced pain and improved health-related QoL and functional disability more than patients receiving usual care, no treatment, or other nonpharmacological interventions [69]. Similarly, Mascarenhas et al [70] found high-quality evidence in favor of CBT for pain in the short term but with a small effect size that did not reach the minimum clinically important change. Although CBT is a common treatment strategy in FM, the studies included in our systematic review did not apply this type of intervention for FM. However, CBT was applied to patients with both IBS and IC/BPS, showing improvements in QoL and functional disability outcomes. Guidelines recommend that the management of these CP conditions should include multimodal behavioral, physical, and psychological techniques [71].

Other self-management interventions delivered by mHealth systems found in the studies included in this review were the monitoring of pain, other symptoms (mood states, disease stages and impact, and adverse events), and PA parameters, isolated or as cointerventions of other therapies. In addition, mind-body components encompassing meditation, mindfulness, and relaxation techniques were found. Nevertheless, the results of these strategies were heterogeneous, showing only some slight differences when compared to usual care or similar intervention by traditional methods. Thus, it suggests that these interventions have insufficient evidence in CP to provide conclusive findings.

Regarding the overall methodological quality of the studies included, almost all of them reported medium methodological quality according to the Checklist for Measuring Quality. Nevertheless, some items related to internal and external validity were frequently scored as “null” or “unable to determine,” which could limit the interpretation and generalization of the results. Likewise, the results of the Cochrane RoB 2.0 assessment tool showed some concerns and a high RoB in the domain related to deviations from intended interventions due to the nature of the study design itself. Lack of blinding of participants is a common issue reported in research where the implementation of interventions depends on the participants, making it difficult to blind them. Similarly, lack of blinding of outcome assessors poses some concerns and a high RoB in the measurement outcome domain, which could also influence the interpretation of findings. Therefore, a future RCT should address these issues to strengthen the evidence on mHealth-based interventions for the self-management of patients with CP.

### Study Limitations and Recommendations for Future Research

Although this systematic review provides a wide perspective on the use of mHealth for self-management of CP, some limitations should be remarked. First, due to the inclusion criteria of the study population, the heterogeneity among CP conditions and patient characteristics makes generalization of the findings not suitable for a specific CP condition. In addition, the high heterogeneity in terms of study interventions and outcome measures makes a meta-analysis not congruent enough to extract a quantitative synthesis of the findings. Third, due to the nature of the RCT, patients in most studies were aware of the interventions, so the effect of a placebo cannot be rejected and could suppose a risk of performance bias. Similarly, the lack of blinding outcome assessors poses a risk of detection bias, which could influence the interpretation of results. Therefore, future research with higher quality in these methodological aspects is needed. Fourth, in some studies, the sample size was small, in addition to losses to follow-up during ongoing research, which could limit the interpretation of the results and limit the drawing of conclusive evidence. Last, because we focused our study on the adult population with CP conditions, the review did not provide information about the effects that the mHealth systems might have on children and adolescents. This could be of interest for future research, as this type of intervention may be attractive and motivating for those populations who are currently familiar with the use of mobile technologies.

### Conclusion

This systematic review analyzed the effects of mHealth systems on self-management interventions in patients with different CP conditions, showing beneficial effects on pain intensity, QoL, and functional disability. Concretely, mHealth systems showed positive effects on pain intensity in CNP, FM, IC/BPS, and OA; on the QoL in CLBP, CNP, IBS, and OA; and on functional disability in CLBP, CMSP, CNP, and OA. No statistically significant changes for any of the study outcomes were observed in patients with unspecific CP and CPP. Despite the methodological limitations, mHealth systems seem to be a promising alternative for the management of patients with CP through a biopsychosocial framework. Indeed, there is a wide variety of mHealth systems for the management of CP, ranging from the monitoring of pain and symptoms to therapeutic approaches, mainly based on exercise, education, and psychosocial components. However, further clinical studies of high methodological quality are needed to consolidate the scientific evidence and recommendations for the use of mHealth systems in patients with CP.

## Appendix

Multimedia Appendix 1 Complete search strategy.

Multimedia Appendix 2 Methodological quality (Checklist for Measuring Quality) and grade of recommendation (OCEBM). OCEBM: Oxford Centre for Evidence-Based Medicine.

Multimedia Appendix 3 mHealth systems used and their features.

## Abbreviations

CBT: cognitive behavioral therapy
CLBP: chronic low back pain
CMSP: chronic musculoskeletal pain
CNP: chronic neck pain
CP: chronic pain
CPG: clinical practice guidelines
CPP: chronic pelvic pain
FIQ: Fibromyalgia Impact Questionnaire
FM: fibromyalgia
HOOS: Hip injury and Osteoarthritis Outcome Score
IBS: irritable bowel syndrome
ICD-11: *International Classification of Diseases 11th Revision*
IC/BPS: interstitial cystitis/bladder pain syndrome
KOOS: Knee injury and Osteoarthritis Outcome Score
mHealth: mobile health
NDI: Neck Disability Index
NRS: numeric rating scale
OA: osteoarthritis
OCEBM: Oxford Centre for Evidence-Based Medicine
PA: physical activity
PRISMA: Preferred Reporting Items for Systematic Reviews and Meta-Analysis
QoL: quality of life
RCT: randomized controlled trial
RoB: risk of bias
SF-36: 36-item Short Form Health Survey
VAS: visual analogue scale
WOMAC: Western Ontario and McMaster
