# Supplementary material for: mHealth Intervention for Improving Pain, Quality of Life, and Functional Disability in Patients With Chronic Pain: Systematic Review
Source: JMIR Mhealth Uhealth. 2023 Feb 2;11:e40844. doi: 10.2196/40844 (PMC9936365; doi:10.2196/40844)
Supplement: Multimedia Appendix 1 [file mhealth_v11i1e40844_app1.pdf]

Multimedia Appendix 1. Complete search strategy.

Step #1 related to chronic conditions	Search (pain* OR "chronic pain" OR "persistent pain" OR ache* OR fibromyalgia OR "chronic migraine" OR "chronic headache" OR lumbago OR lumbalgia OR dorsalgia OR "irritable bowel syndrome" OR "temporomandibular disorder pain" OR "burning mouth syndrome" OR osteoarthritis OR spondylosis OR neuropath* OR "trigeminal neuralgia" OR "tolosa-Hunt syndrome")
Step #2 related to mHealth interventions	Search (mhealth OR "m-health" OR smartphone OR mobile* OR iphone OR ipad) (app OR apps OR application*)
Step #5 related to mHealth interventions	Search (app OR apps OR application*)
Step #4 related to study design	Search (“randomized controlled trial*” OR “randomised controlled trial*” OR “controlled clinical trial*” OR “randomized clinical trial*” OR “randomised clinical trial” OR “clinical trial*”)
Step #5	Search (#1 AND #2 AND #3 AND #4)
