# Supplementary material for: mHealth Intervention for Improving Pain, Quality of Life, and Functional Disability in Patients With Chronic Pain: Systematic Review
Source: JMIR Mhealth Uhealth. 2023 Feb 2;11:e40844. doi: 10.2196/40844 (PMC9936365; doi:10.2196/40844)
Supplement: Multimedia Appendix 3 [file mhealth_v11i1e40844_app3.pdf]

Multimedia Appendix 3. mHealth[a] systems used and their features.

CP[b] condition and mHealth app		Purpose	Main features
**CP**			
	MORPH Companion [43]	Monitoring symptoms and healthy behaviors	The app provides both adaptative ecological strategies and assessments to reduce patients' burden and pain episodes. It is an intervention designed to incite to be active in daily life, and to promote social influences and relationships, as well as the patients' expectations and their ability to be self-efficient. In addition, there are available telecoaching sessions about sedentary behavior and weight loss on the app.
	Fitbit [43]	Monitoring PA[c] parameters	Among all the features available of this commercial app, it is used for exercise monitoring and tracking daily activity (daily and long-term progress in the number of steps, calorie goals, and active time).
**CMSP[d]**			
	Pain Monitor [38]	Monitoring symptoms	The app evaluates 34 pain-related variables twice a day (once in the morning and once in the afternoon, at configurable hours). Moreover, it allows patients to record acute pain episodes, monitoring them in real time.
**CLBP[e]**			
	Snapcare [53]	Monitoring pain-related outcomes and PA parameters<br>Home-based PA program	The app collects daily PA parameters, displays day-to-day activity goals, and includes a set of prescribed home-based exercise and aerobic exercises. Feedback is available at the end of each PA session, using a system of rewards to enhance engagement. The app allows the patient to modify and adjust the goals based on their health status, activities in daily living, daily PA performance, and functional progress.
	IMPACT [52]	Monitoring PA parameters	The app allows patients to enter their PA goals and activities performed in daily routines.
	Pain Care [41]	Monitoring pain-related outcomes and PA parameters	The app allows patients to enter pain episodes and intensity, and data related to their PA levels. In addition, patients can retrieve their data and adapt the setting of the reminders for requesting these data.
	FitBack [47]	Monitoring pain-related outcomes<br>CBT[f] and education	The app includes 30 videos clips on basic aspects of pain and self-management, cognitive and behavioral approaches (mindfulness and relaxation, and the importance of being physically active, controlling fear beliefs) and videos clips on specific strength and stretching exercises. It also tracks the frequency and level of pain in each episode and the daily self-care activities used to manage pain. Weekly emails and messages can also be sent by the app as reminders to track pain and self-care activities.
**The CNP[g]**			
	NeckProtector [40]	Monitoring pain-related outcomes and PA parameters<br>Home-based PA program	According to a previous breathing test through the app and the patient's pattern, it chooses the appropriate number of exercises sessions. The app encompasses an exercise module, including 5 postures for each session, and records of pain intensity, before and after exercise, and the painful areas; a statistical menu, which shows the summarizing graphs of daily and monthly exercise results; and an education module with materials about the causes of neck pain and how to handle pain.
	Smartphone app [49]	Monitoring pain-related outcomes and PA parameters	The app provides McKenzie neck exercise programs, depending on the type of neck pain identified. It offers a self-feedback feature for patients to follow their

		Home-based PA program	accumulative exercise records and changes in the level of pain.
	Seeb [51]	Home-based PA program<br>Monitoring PA parameters	The app includes the descriptions of PA programs (type of exercise, repetitions, and a picture of each exercise) and the self-managed postures on work time, the record of administration instruction and the user reaction to the warning, and the exercise administration schedule. It also sends alarms, which can be predetermined depending on the work time and the PA program.
	Relaxneck [54]	Relaxation techniques, mindfulness training, and CBT[f] strategies	The app includes 3 types of relaxation exercises (autogenic training, mindfulness meditation, and guided imagery). These exercises can be performed in different positions according to the patient's needs. In addition, a feature based on CBTs was implemented in the app for the study intervention and also supports notification features, a diary, and questionnaires.
**FM[h]**			
	PASS Clinic [48]	Monitoring symptoms	The app is part of the Pain Assessment and Analysis System (PAAS), which aims to track data related to pain severity and environmental factors. The app summarizes pain data through graphs based on daily, weekly, or monthly periods. It also has a feature to record the time, temperature, humidity, and weather when pain occurs to determine any correlations between pain episodes and some environmental factors.
	ProFibro [42]	Monitoring the FIQ[i] domains<br>Education and home-based PA program	The app includes educational materials; self-monitoring with the FIQ; graded PA programs involving aerobic, stretching, and strengthening exercises; sleep strategies with a guided imagery relaxation technique, stimulus control therapy, and sleep hygiene; a scheduling function for daily activity planning; a diary for the practice of gratitude; and material for family adjustment and recommendations through notifications. In addition, the app allows the patient to self-monitor the disease impact and health-related outcomes with the FIQ.
**OA[j]**			
	OA GO [37]	Monitoring symptoms and PA-related goals	The app requests patients to record information about pain and mood states. In addition, it gives motivational messages to encourage patients to achieve their daily step goals, and it shows daily and monthly activity trends.
	My Dear Knee [33]	Monitoring PA parameters<br>Home-based PA program	The app includes prescribed PA programs (type of exercise, repetitions, and preferred scheduled time) using colorful and animated images. It records exercise adherence and automatically sends alarms and notifications for each exercise session.
	Dr. Bath [35]	Monitoring PA-related goals<br>Education and home-based PA program	The app is divided into an educational menu, which includes information about OA management and its treatment modalities, and recommendations promoting a healthy lifestyle, and an exercise module, including specific exercises related to OA treatment. It proposes preformulated goals to patients, depending on the personal profile and preferences and uses motivational strategies to reinforce adherence and health-related behaviors, including reminders and rewards.
	HereAfter [45]	Monitoring PA parameters<br>Education and home-based PA program	The app contains PA programs based on neuromuscular leg strengthening, core stability, and performance and balance enhancement. It also provides educational sessions, including basic OA information, its treatment modalities, self-managing symptoms, and the importance of behavioral changes and maintaining a healthy lifestyle. The app sends daily emails or notifications to encourage adherence.

	Rak Kao [39]	Monitoring symptoms Education and home-based PA program	The app has 4 modules, including (1) knowledge of the disease, its ongoing, and symptoms; (2) treatment strategies; (3) a personalized approach according to the OA stage and severity; and (4) exercise instructions and recommendations. It contains a previous assessment to determine the OA stage, and depending on it, the app recommends a suitable type of exercises and repetitions and brief information about symptom self-management.
	Smartphone app [34]	Education	The app provides educational information related to basic OA knowledge and its causes, potential risk factors, healthy diet habits, different treatment approaches, and exercises prescribed for the OA condition.
**IBS[k]**			
	AI Heali [36]	Nutrition information and dietary plans	The app can scan menus and barcodes to give nutritional information and advice according to patient-specific diet plans and patients' preferences. It also contains a traffic light system to allow the patient to match the type of foods according to the nutritional information.
	Zemedy [46]	CBT	The app contains a total of 10 modules based on psychoeducation, CBT strategies involving relaxation training, exercises, cognitive strategies to manage stress states and catastrophizing, exposure exercises for reducing avoidance, behavioral experiments, and healthy nutritional recommendations.
**CPP[l]**			
	The Headspace [44]	Mindfulness meditation training	The app teaches mindfulness with autoguided meditation, with a 10-day basic meditation module followed by a specific pain module. It also provides basic information and training in mindfulness meditation aimed at CP.
**IC/BPS[m]**			
	TICA [50]	Monitoring symptoms, QoL[n], and lifestyle routine Education and self-management	The app contains 21 video clips, which provide education health, including diet recommendations, encouragements to perform PA, meditation and yoga sessions, and hygiene and sexual advice. It also contains a menu for self-management of emergent symptoms flare, with a list of questions related to pain experiences and possible causes. In addition, it records the patient's diets and life routines every week and tracks the study assessment questionnaires (QoL, pain intensity, and symptom data).

[a]mHealth: mobile health.
[b]CP: chronic pain.
[c]PA: physical activity.
[d]CMSP: chronic musculoskeletal pain.
[e]CLBP: chronic low back pain.
[f]CBT: cognitive behavioral therapy.
[g]CNP: chronic neck pain.
[h]FM: fibromyalgia.
[i]FIQ: Fibromyalgia Impact Questionnaire.
[j]OA: osteoarthritis.
[k]IBS: irritable bowel syndrome.
[l]CPP: chronic pelvic pain.
[m]IC/BPS: interstitial cystitis/bladder pain syndrome.
[n]QoL: quality of life.
